# Higher skin carotenoid levels are associated with lower risks of metabolic syndrome: a cross-sectional study in Vietnamese participants

**DOI:** 10.3389/fnut.2025.1715158

**Published:** 2026-01-22

**Authors:** Kazutaka Yoshida, Yuichiro Nakazawa, Thuy Lan Nguyen, Duc Huy Nguyen, Chu Xuan Anh, Shingo Takahashi, Shigenori Suzuki, Vu Quoc Binh

**Affiliations:** 1 Diet & Well-being Research Institute, Kagome Co., Ltd., Nasushiobara, Japan; 2 Dr. Binh Tele_Clinic – Branch of E2E Solution Company Limited, Hanoi, Vietnam

**Keywords:** carotenoid, cross-sectional study, metabolic syndrome, skin carotenoid level, Vietnamese

## Abstract

**Introduction:**

Metabolic syndrome (MetS) is a major health concern in Vietnam. Although carotenoids have been suggested to suppress MetS, there has been no research on the relationship between carotenoid levels in the body and MetS risk in Vietnam. Therefore, this study clarified the relationship between skin carotenoid levels and markers related to MetS in Vietnamese adults using Vegecheck®, a device for non-invasively measuring skin carotenoid levels.

**Methods:**

This cross-sectional study included 300 participants, and data were collected at the Dr. Binh TeleClinic, a hospital in Hanoi, Vietnam. Data on skin carotenoid levels, anthropometric parameters (height, weight, and body fat percentage), blood pressure, and blood parameters (glucose, triglyceride, and high- and low-density lipoprotein cholesterol levels) were collected. MetS risk counts were defined as the number of MetS components in accordance with international standards for MetS. Additionally, participants answered questions regarding their attributes and lifestyles using a simple questionnaire.

**Results:**

After excluding 22 participants with missing data, 278 participants were included in the analysis. The average skin carotenoid levels in all participants were 5.41 ± 1.31, 4.99 ± 1.10 in men, and 5.67 ± 1.37 in women. Higher skin carotenoid levels were associated with lower MetS risk counts. The odds ratio (OR), with 95% confidence interval (CI), was 0.767 [0.645–0.910] (*p* = 0.003). Skin carotenoid levels are significantly negatively associated with body weight, body mass index, blood glucose, and triglyceride levels. The estimated regression coefficient and 95% CI after multivariable adjustment were −0.016 [−0.0301, −0.0011] (*p* = 0.034), −0.018 [−0.0305, −0.0045] (*p* = 0.008), −0.043 [−0.0686, −0.0177] (*p* < 0.001), and −0.090 [−0.1429, −0.0374] (*p* < 0.001), respectively.

**Conclusion:**

This study indicates a relationship between carotenoid levels and MetS risk in Vietnamese individuals, although a causal relationship between carotenoid intake and MetS could not be established. This study has some limitations, such as the influence of unknown confounding factors and selection bias, and the unclearness of individual carotenoids’ effects. Future observational and intervention studies taking these limitations into account are required.

**Clinical trial registration:**

ClinicalTrials.gov, identifier NCT06271070.

## 1 Introduction

The proportion of deaths from non-communicable diseases has increased from 58.1 to 68.3% globally between 1990 and 2020, and the situation is worse in Vietnam, where it rose from 58.0 to 79.8% (1); hence, the prevention of lifestyle-related diseases such as cancer, cardiovascular disease, and diabetes is an important health issue in Vietnam. Metabolic syndrome (MetS) is the main cause of lifestyle-related diseases (2). A meta-analysis of 18 studies conducted in Vietnam reported that MetS prevalence was 16.1% among 35,421 healthy adults aged <65 years (3). Additionally, a survey of 300 companies in Vietnam reported that 16% of employees aged ≥18 years, 34.6% of those in their 50s, and 49.1% of those aged >60 years had MetS (4). MetS is an important health issue in Vietnam, particularly among middle-aged and older adults.

Carotenoids are pigment components with antioxidant capacities that are mainly found in vegetables, fruits, and algae, and have various health benefits, such as preventing cancer and cardiovascular disease, and improving cognitive and visual functions (5). As oxidative stress is one of the causes of MetS, carotenoids are suggested to have a suppressive effect on MetS (6, 7). Several studies have shown a significant relationship between blood carotenoid concentration, MetS prevalence, and MetS biomarker levels (6–12). However, there have been no studies on the relationship between carotenoid intake or carotenoid levels and MetS prevalence and/or MetS biomarker levels in Vietnam.

Clarifying the relationship between carotenoid levels in the body and MetS prevalence and/or MetS biomarker levels in Vietnamese individuals is essential, as this can inform dietary strategies to prevent MetS in Vietnam. However, measuring blood carotenoid concentrations is invasive and requires both time and financial resources. Vegecheck® was developed as a device for non-invasively measuring skin carotenoid levels (13); it uses multiple spatially resolved reflection spectroscopy (MSRRS) sensors (14–16). The MSRRS method can measure skin carotenoid levels more easily and at a lower cost than the conventional and highly accurate resonance Raman spectroscopy (RRS) method; however, the measurement results obtained by the MSRRS method have a strong correlation with those obtained by RRS (15, 16). The MSRRS sensor irradiates a light-emitting diode (LED) on the palm of the hand, and the reflected light obtained from various depths and angles is measured by a detection unit inside the sensor, and carotenoid levels are calculated using a unique algorithm (14). This method can reduce the influence of chromophores such as melanin, flavin adenine dinucleotide (FAD), reduced FAD, deoxy-hemoglobin, or oxy-hemoglobin and bilirubin, which are mostly distributed in the basal level and in the dermis of the skin (14, 16). The MSRRS sensor was developed by calibration to the RRS device using more than 3,000 measurements, and a special algorithm was established to correct production tolerances of the MSRRS sensor (16). The MSRRS method can produce adequate results independent of the skin type of the subjects because the correlation between skin carotenoid levels and blood carotenoid concentrations in Fitzpatrick skin types I–III measured by the MSRRS method is similar to that in Fitzpatrick skin types IV–VI (16).

Some cross-sectional studies in Japanese participants have reported that skin carotenoid levels measured by Vegecheck® are significantly associated with blood carotenoid concentrations, vegetable intake, MetS prevalence, and MetS biomarker levels (17–19). Therefore, a cross-sectional study was conducted to clarify the relationship between skin carotenoid levels measured by Vegecheck® and biomarker levels related to MetS. Verifying whether the findings previously observed in the Japanese population also apply to Vietnamese individuals could provide important insights into the generalizability of the relationship between skin carotenoid levels and MetS.

## 2 Materials and methods

### 2.1 Ethical review and protocol registration

This study was approved by the E2E Solution Medical Centre IRB #1 - Biomedical (protocol number: 2023-R23, approval date: January 1, 2024), a third party not involved in the study. The study was conducted in accordance with the World Medical Association Declaration of Helsinki, International Conference on Harmonization Guidelines for Good Clinical Practice (ICH E6R2), and Ethical Guidelines for Medical and Health Research Involving Human Subjects, which were established by the Japanese government to ensure ethical conduct in human research. The study protocol was registered at ClinicalTrials.gov (ID number: NCT06271070) on January 30, 2024.

### 2.2 Study design

This cross-sectional study was conducted at Dr. Binh Teleclinic (Hanoi, Vietnam), and the study participants were recruited from visitors to the clinic. Written informed consent was obtained from all participants before data and blood collection. The participants fasted from breakfast on the day of data and blood collection (only water or warm water was permitted). Each participant attended the clinic for a single visit during the study period, during which skin carotenoid levels, anthropometric measurements, blood pressure, and questionnaire responses were collected, along with a 2 mL blood sample. Blood parameters were measured at the clinic. The data and blood samples were collected intermittently between January 30 and April 16, 2024. This study adhered to the Strengthening the Reporting of Observational Studies in Epidemiology (STROBE) guidelines (20).

### 2.3 Participants

The inclusion criteria were as follows: (1) adults aged ≥18 years; (2) healthy individuals or patients with metabolic diseases such as obesity, diabetes, hypertension, lipid abnormalities, and hyperuricemia; and (3) those who agreed to participate in the study and were able to complete the informed consent form.

The exclusion criteria were as follows: (1) hospitalized patients; (2) patients with diseases other than metabolic diseases, as written in the inclusion criteria; and (3) people judged to be inappropriate by the principal investigator.

Three hundred people who met the eligibility criteria and agreed to participate in the study with written informed consent were selected as study participants.

### 2.4 Sample size calculation

Sample size calculation based on previous studies conducted in Vietnam was not feasible, as no prior study has analyzed the correlation between skin carotenoid levels and the risk and/or biomarker levels of MetS in Vietnam.

In a cross-sectional study conducted in Japan (18), higher skin carotenoid levels were significantly associated with lower body mass index (BMI), systolic blood pressure (SBP), diastolic blood pressure (DBP), blood triglyceride levels, and high blood high-density lipoprotein cholesterol (HDL-C) levels in women (*n* = 471) and significantly correlated with BMI in men (n = 340). In another unpublished cross-sectional study conducted in Japan, higher skin carotenoid levels were significantly associated with lower BMI, SBP, DBP, blood triglyceride levels, and higher blood HDL-C levels in women (*n* = 283), and significantly associated with lower BMI, SBP, DBP, and blood triglyceride levels in men (*n* = 109). Based on these data, we targeted at least 100 participants to observe a significant correlation between skin carotenoids and biomarker levels related to MetS. In addition, the MSRRS method used for the detection of skin carotenoid by Vegecheck® is reported to produce adequate results independent of the skin type (16). Additionally, carotenoid intake is almost similar among Vietnamese and Japanese individuals (21, 22). Based on these reports, we assumed that the skin carotenoid levels in Vietnamese people are almost similar to those of Japanese people. Therefore, the sample size (*n* = 300) was considered sufficient to achieve the study objectives.

### 2.5 Data collection

#### 2.5.1 Skin carotenoid level

Skin carotenoid levels were measured using Vegecheck® (Kagome Co., Ltd., Nagoya, Japan), which uses an MSRRS sensor (Biozoom Services GmbH, Kassel, Germany) as mentioned in previous studies (17–19). The sensor utilized LED light emitters to provide light within the range of 350–1,000 nm, while detecting the reflected light from light-sensitive areas of the skin (16). To ensure the best possible correlation with the measured RRS values, an algorithm was developed to calculate the rank-order skin carotenoid score, which ranged from 0.1 to 12.0. Skin carotenoid level was measured by placing the palm of the hand over the sensor, which was completely covered so that no stray light could enter. It was remeasured if any error messages were displayed.

#### 2.5.2 Anthropometric parameters and blood pressure

Height, weight, and BMI were measured using an automatic portable stadiometer BSM370 (InBody, Co., Ltd., Seoul, Korea). Body fat percentage was measured using a portable body composition analyzer, InBody270 (InBody Co., Ltd.). The SBP and DBP were measured using an automatic blood pressure monitor JPN750 (Omron Co., Ltd., Kyoto, Japan).

#### 2.5.3 Blood parameters

The concentrations of blood glucose, triglycerides, low-density lipoprotein cholesterol (LDL-C), and HDL-C were measured using an AU480 Chemistry Analyzer (Beckman Coulter, Inc., CA, USA).

#### 2.5.4 Questionnaire

The questionnaire included questions about sex, age, monthly income, academic background, medication, diet therapy, and lifestyle behavior (smoking, drinking, vegetable intake). The questionnaire (English version) is shown in Supplementary Data Sheet 1.

### 2.6 Statistical analysis

#### 2.6.1 Assessment of MetS risks

The criteria for MetS (obesity/overweight, elevated blood pressure, elevated fasting blood glucose, elevated blood triglycerides, and reduced blood HDL-C) were set in accordance with international standards (23, 24). Obesity/overweight was defined as BMI ≥ 25 kg/m^2^. Elevated blood pressure was defined as SBP ≥ 130 mmHg and/or DBP ≥ 85 mmHg and/or taking antihypertensive medication. Elevated fasting blood glucose was defined as fasting blood glucose level ≥110 mg/dL and/or taking antidiabetic drugs. Elevated blood triglyceride was defined as fasting blood triglyceride level ≥150 mg/dL or taking antihyperlipidemic drugs. Reduced blood HDL-C was defined as blood HDL-C level <40 mg/dL for men and <50 mg/dL for women. The number of MetS components judged in accordance with the above criteria was defined as the MetS risk count on a six-level scale, with the lowest being zero and the highest being five.

#### 2.6.2 Regression analyses

Categorical variables were set as follows for regression analyses: sex, female = 0 and male = 1; academic background, other than university or graduate school = 0 and university or graduate school = 1; current smoking status, non-smoking = 0 and smoking = 1; current drinking status, non-drinking = 0 and drinking = 1; and monthly income, <10 million VND = 0 and ≥ 10 million VND = 1.

The relationship between MetS risk counts and factors related to MetS was analyzed using ordinal logistic regression analysis, with MetS risk counts (zero–five) as the objective variable and sex, academic background, current smoking and drinking status, monthly income, age, and skin carotenoid level as explanatory variables. To assess whether the proportional odds assumption of the ordinal logistic regression model was satisfied, we conducted the Brant test. The Brant test evaluates parallel regression assumption, and a non-significant test result indicates that the proportional odds assumption holds (25).

The relationship between skin carotenoid levels and anthropometric parameters, blood pressure, and blood parameters was analyzed using multiple regression analysis, with the parameters as the objective variables and skin carotenoid level as the explanatory variable. Sex, academic background, current smoking and drinking status, monthly income, and age were used as moderating variables in other studies evaluating the correlation between skin or blood carotenoid levels and MetS prevalence (10, 19). Anthropometric parameters, blood pressure, and other blood parameters were converted to natural logarithms for multiple regression analysis because they did not follow a normal distribution. We assessed multicollinearity among the variables using the variance inflation factor (VIF). A VIF value greater than 10 was considered indicative of potentially problematic multicollinearity. The overall significance of the regression model was evaluated using the F-statistic and its corresponding *p*-value.

EZR (Ver. 1.61) (26) was used for all statistical analyses.

## 3 Results

### 3.1 Flow diagram

**Figure 1:**
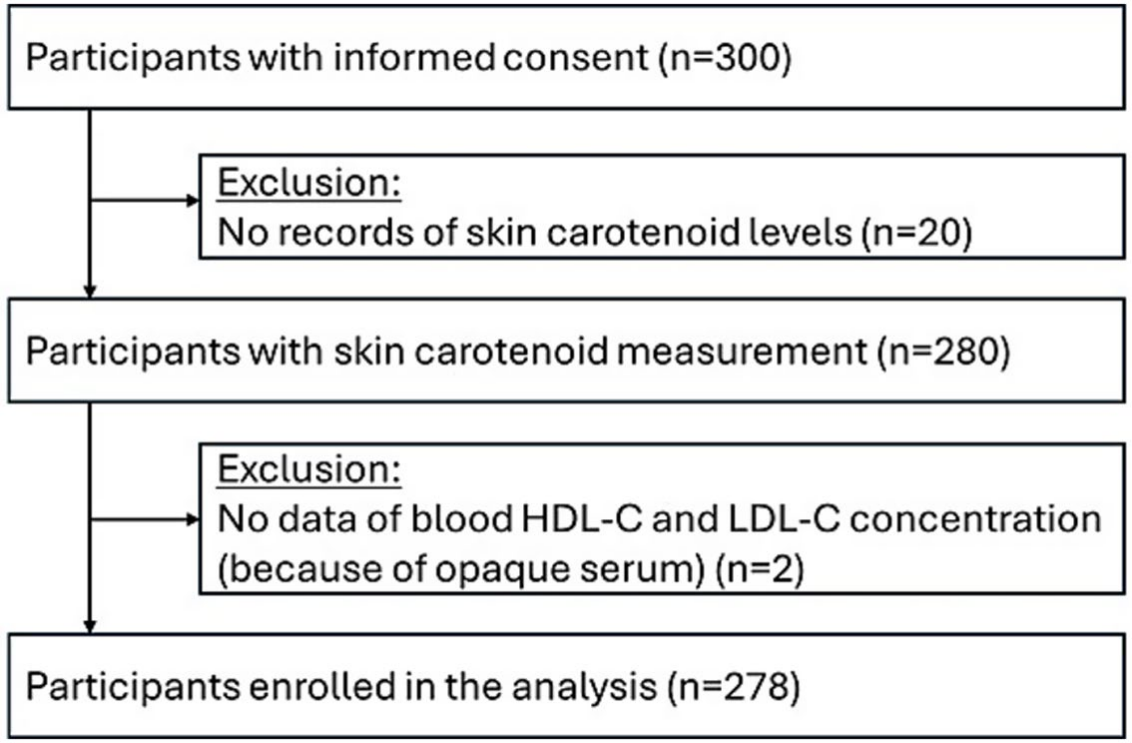
Flow diagram for the study participants.

Data were collected from 300 participants who met the eligibility criteria and agreed to participate in the study after providing written informed consent. Of the 300 participants, data on skin carotenoid levels were not accurately collected for 20 participants, and blood LDL-C and HDL-C concentrations could not be measured for two participants because of their opaque serum. Data from the remaining 278 participants were analyzed (Figure 1). No adverse events were reported during the study period.

### 3.2 Participant characteristics

**Figure 2:**
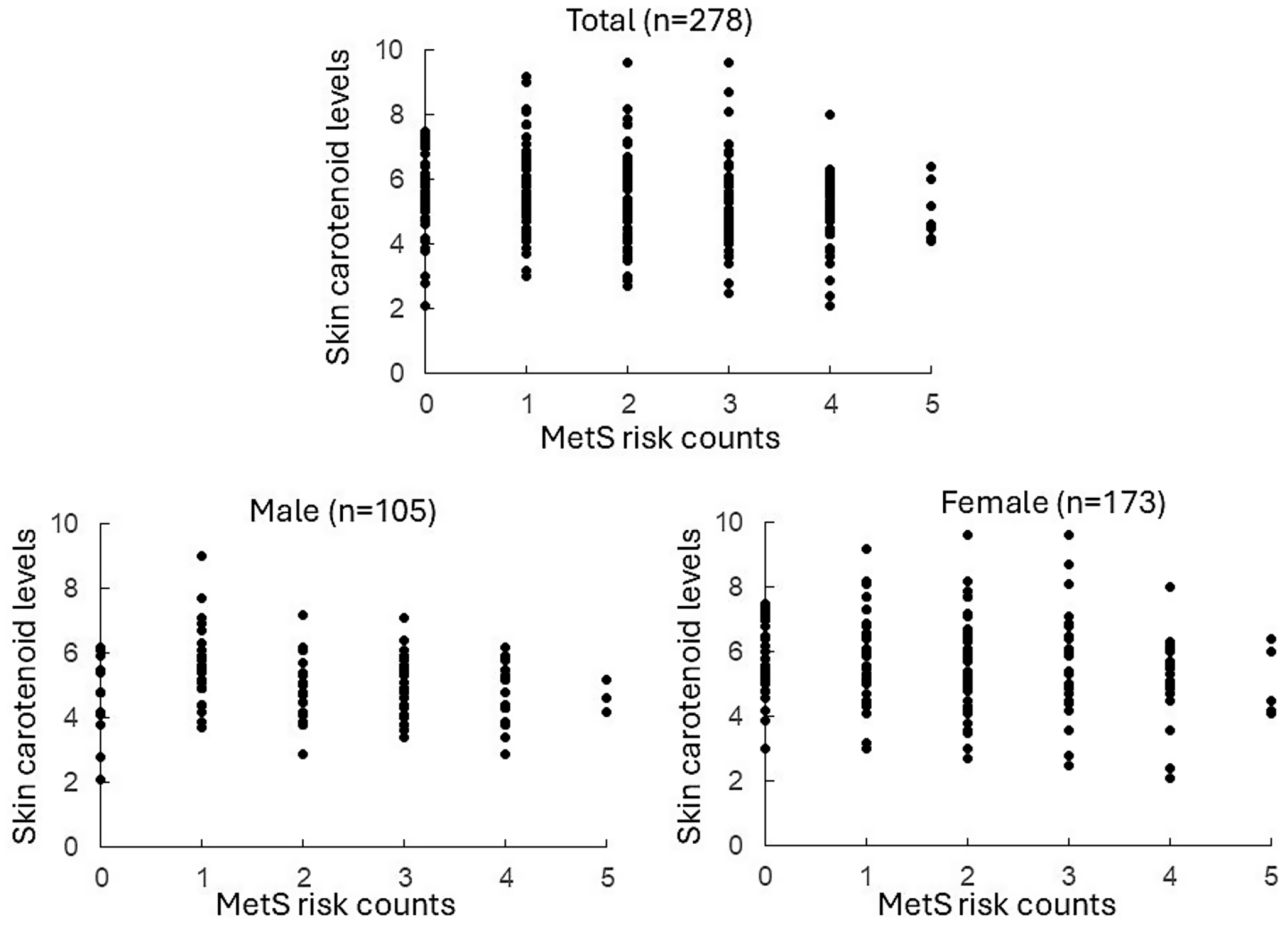
Scatter plot of skin carotenoid levels and MetS risk counts for all subjects and for each sex. The x-axis shows the distribution of MetS risk counts. The y-axis shows the distribution of skin carotenoid levels.

Table 1 shows the number of participants with each MetS component. Of the 278 participants analyzed, 29.5% were classified as obese or overweight, 59.7% had elevated blood pressure, 41.0% had elevated fasting blood glucose levels, 44.6% had elevated blood triglyceride levels, and 25.5% had reduced blood HDL-C levels. Table 2 shows the participants’ characteristics stratified by MetS risk counts. Participant characteristics by sex are shown in Supplementary Table 1. The mean skin carotenoid level was 5.41 ± 1.31 for all participants (*n* = 278), 4.99 ± 1.10 for males (*n* = 105), and 5.67 ± 1.37 for females (*n* = 173). The scatter plot of skin carotenoid levels and MetS risk counts for all subjects and for each sex is shown in Figure 2.

**Table 1:** Number of participants with each MetS component.

MetS components	Criteria	Applicable	Not-applicable
Overweight/obesity	BMI ≥ 25.0 kg/m^2^	82 (29.5%)	196 (70.5%)
Elevated blood pressure	SBP ≥ 130 mmHg and/or DBP ≥ 85 mmHg and/or antihypertensive drug treatment	166 (59.7%)	112 (40.3%)
Elevated fasting blood glucose	Fasting blood glucose≥110 mg/dL and/or antidiabetes drug treatment	114 (41.0%)	164 (59.0%)
Elevated blood triglyceride	Blood triglyceride≥150 mg/dL and/or antihyperlipidemia drug treatment	124 (44.6%)	154 (55.4%)
Reduced blood HDL-C	<40 mg/dL (Male), < 50 mg/dL (Female)	71 (25.5%)	207 (74.5%)

BMI, body mass index; SBP, systolic blood pressure; DBP, diastolic blood pressure; HDL-C, high-density lipoprotein cholesterol.

**Table 2:** Participant characteristics for each MetS risk count.

Factors	Total	0	1	2	3	4	5
Number of subjects	278	53	62	59	52	44	8
Male/Female	105/173	13/40	25/37	20/39	24/28	20/24	3/5
Current smoking^1^	40 (14.4%)	5 (9.4%)	6 (9.7%)	5 (8.5%)	9 (17.3%)	12 (27.3%)	3 (37.5%)
Alcohol intake^1^	61 (21.9%)	6 (11.3%)	15 (24.2%)	11 (18.6%)	19 (36.5%)	9 (20.5%)	1 (12.5%)
Medication^1^	146 (52.5%)	4 (7.5%)	18 (29.0%)	37 (62.7%)	39 (75.0%)	41 (93.2%)	7 (87.5%)
Vegetable intake^2^	141 (50.7%)	28 (52.8%)	36 (58.1%)	22 (37.3%)	29 (55.8%)	24 (54.5%)	2 (25.0%)
Monthly income^3^	154 (55.4%)	36 (67.9%)	43 (69.4%)	26 (44.1%)	22 (42.3%)	22 (50.0%)	5 (62.5%)
Academic background^4^	112 (40.3%)	39 (73.6%)	38 (61.3%)	12 (20.3%)	15 (28.8%)	5 (11.4%)	3 (37.5%)
Skin carotenoid level	5.41 ± 1.31	5.58 ± 1.24	5.76 ± 1.33	5.35 ± 1.41	5.32 ± 1.38	4.98 ± 1.11	4.90 ± 0.88
Age (years)	51.83 ± 15.53	36.92 ± 12.91	46.58 ± 16.15	57.25 ± 13.09	58.87 ± 11.91	61.09 ± 8.36	54.75 ± 7.32
Height (cm)	158.43 ± 8.18	159.36 ± 6.94	159.71 ± 7.77	157.08 ± 9.52	158.52 ± 8.49	157.11 ± 7.91	159.00 ± 7.50
Weight (cm)	59.79 ± 11.67	53.06 ± 6.24	58.10 ± 8.96	58.79 ± 10.98	61.89 ± 10.31	66.10 ± 13.95	76.39 ± 21.41
BMI (kg/m^2^)	23.76 ± 3.71	20.90 ± 2.16	22.70 ± 2.49	23.67 ± 2.74	24.51 ± 2.75	26.79 ± 4.03	30.13 ± 7.64
Body fat percentage (%)	25.55 ± 8.76	24.84 ± 7.60	23.53 ± 8.68	26.20 ± 9.03	23.55 ± 7.26	29.33 ± 8.26	33.15 ± 15.62
SBP (mmHg)	128.81 ± 18.47	110.83 ± 9.75	123.34 ± 16.27	136.08 ± 17.51	137.46 ± 16.44	136.25 ± 16.54	139.50 ± 10.58
DBP (mmHg)	78.68 ± 11.69	67.98 ± 7.35	75.08 ± 10.86	81.29 ± 9.93	83.67 ± 10.64	85.34 ± 9.96	89.13 ± 9.91
Blood glucose (mg/dL)	115.57 ± 48.53	89.17 ± 6.49	98.68 ± 12.92	111.95 ± 37.86	136.13 ± 64.26	142.17 ± 65.35	168.20 ± 67.50
Blood triglyceride (mg/dL)	162.11 ± 121.97	73.87 ± 28.96	111.82 ± 57.74	166.55 ± 124.38	200.79 ± 118.98	254.58 ± 130.48	343.55 ± 183.18
Blood LDL-C (mg/dL)	114.31 ± 31.36	102.43 ± 23.11	116.51 ± 36.28	116.49 ± 29.02	117.21 ± 29.35	117.01 ± 35.87	126.34 ± 29.68
Blood HDL-C (mg/dL)	55.00 ± 13.87	63.08 ± 14.13	58.68 ± 13.06	55.16 ± 11.21	52.96 ± 13.81	45.17 ± 9.98	39.24 ± 7.95

1: Number of “Yes”, 2: Number of “Eat almost every day”, 3: Number of “≧10 million VND/month”, 4: Number of “University” or “Graduate school”.

BMI, body mass index; SBP, systolic blood pressure; DBP, diastolic blood pressure; LDL-C, low-density lipoprotein cholesterol; HDL-C, high-density lipoprotein cholesterol.

The values are expressed as means ± S. D.

### 3.3 Relationship between MetS risk counts and skin carotenoid levels

#### 3.3.1 Relationship between the MetS risk counts and each factor

Table 3 shows the results of the ordinal logistic regression analysis. Skin carotenoid levels, age, and academic background were independent variables that were significantly associated with MetS risk counts. The odds ratios and 95% confidence intervals (CI) were 0.767 [0.645–0.910], 1.070 [1.050–1.090], and 0.533 [0.288–0.981] for skin carotenoid levels, age, and academic background, respectively. The result of the Brant test indicated that these variables met the proportional odds assumption (*p* = 0.41, *p* = 0.09, *p* = 0.06, respectively).

**Table 3:** Relationship between MetS risk counts and each factor.

Explanatory variables	OR	95% CI	*p*-value
Sex	1.600	[0.881, 2.900]	0.123
Academic background	0.533	[0.288, 0.981]	0.044*
Current smoking	2.020	[0.962, 4.230]	0.063
Current drinking	0.933	[0.468, 1.860]	0.845
Monthly income	1.210	[0.751, 1.960]	0.431
Age	1.070	[1.050, 1.090]	<0.001***
Skin carotenoid level	0.767	[0.645, 0.910]	0.003**

Ordinal logistic regression analysis was performed with the following factors: age, sex, academic background, current smoking, current drinking, monthly income, and skin carotenoid levels.

Categorical variables were set as follows for regression analyses: sex, female = 0 and male = 1; academic background, other than university or graduate school = 0 and university or graduate school = 1; current smoking, non-smoking = 0 and smoking = 1; current drinking, non-drinking = 0 and drinking = 1; and monthly income, <10 million VND = 0 and ≥ 10 million VND = 1.

An odds ratio of an explanatory variable greater than one means that the factor increases MetS risk. An odds ratio of an explanatory variable less than one means that the factor decreases MetS risk counts.

OR, odds ratio; CI, confidence interval.

* *p* < 0.05, ** *p* < 0.01, *** *p* < 0.001.

#### 3.3.2 Relationship between skin carotenoid levels and anthropometric parameters, blood pressure, and blood parameters

Table 4 shows the results of the multiple regression analysis. In the unadjusted model, skin carotenoid levels showed a significant negative association with height, weight, BMI, blood glucose, and triglyceride levels, and a significant positive association with HDL-C. Since the results of participant characteristics suggested that age and sex may affect the relationship between skin carotenoid levels and MetS biomarkers, age- and sex-adjusted analysis was conducted. In addition, multivariate-adjusted analysis was also performed, including representative moderating variables that have been previously reported in previous studies. In the age- and sex-adjusted, and multivariate-adjusted models, skin carotenoid levels showed a significant negative association with weight, BMI, blood glucose, and triglyceride levels. The estimated regression coefficient and 95% CI after multivariable adjustment were −0.016 [−0.0301, −0.0011], −0.018 [−0.0305, −0.0045], −0.043 [−0.0686, −0.0177], and −0.090 [−0.1429, −0.0374], respectively. Skin carotenoid levels showed a marginally significant positive association with HDL-C levels (0.020 [−0.0022, 0.0417]). The multiple regression model for these valuables was statistically significant [*F*(7, 270) = 8.47, *p* < 0.001 for weight; *F*(7, 270) = 4.93, *p* < 0.001 for BMI; *F*(7, 270) = 8.82, *p* < 0.001 for blood glucose; and *F*(7, 270) = 11.03, *p* < 0.001 for blood triglyceride]. The VIF value indicated no potentially problematic multicollinearity.

**Table 4:** Relationship between skin carotenoid levels and anthropometric parameters, blood pressure, and blood parameters.

Parameters	Unadjusted			Age- and sex-adjusted			Multivariable-adjusted		
	RC	95% CI	*p*-value	RC	95% CI	*p*-value	RC	95% CI	*p*-value
Height	−0.006	[−0.0110, −0.0018]	0.006**	0.001	[−0.0029, 0.0040]	0.765	0.001	[−0.0027, 0.0042]	0.662
Weight	−0.033	[−0.0487, −0.0171]	<0.001***	−0.017	[−0.0312, −0.0022]	0.024*	−0.016	[−0.0301, −0.0011]	0.034*
BMI	−0.020	[−0.0327, −0.0071]	0.002**	−0.018	[−0.0306, −0.0050]	0.007**	−0.018	[−0.0305, −0.0045]	0.008**
BFP	−0.005	[−0.0372, 0.0263]	0.736	−0.018	[−0.0497, 0.0133]	0.257	−0.019	[−0.0506, 0.0130]	0.246
SBP	−0.003	[−0.0162, 0.0097]	0.619	−0.003	[−0.0140, 0.0080]	0.589	−0.002	[−0.0135, 0.0086]	0.666
DBP	−0.012	[−0.0258, 0.0013]	0.077	−0.011	[−0.0228, 0.0014]	0.083	−0.010	[−0.0217, 0.0026]	0.124
Glucose	−0.037	[−0.0637, −0.0109]	0.006**	−0.044	[−0.0690, −0.0183]	<0.001***	−0.043	[−0.0686, −0.0177]	<0.001***
Triglyceride	−0.104	[−0.1590, −0.0483]	<0.001***	−0.102	[−0.1548, −0.0485]	<0.001***	−0.090	[−0.1429, −0.0374]	<0.001***
LDL-C	−0.012	[−0.0373, 0.0140]	0.374	−0.018	[−0.0444, 0.0080]	0.172	−0.016	[−0.0427, 0.0102]	0.228
HDL-C	0.035	[0.0129, 0.0574]	0.002**	0.022	[−0.0000, 0.0439]	0.050	0.020	[−0.0022, 0.0417]	0.078

Multiple regression analysis was performed with each parameter as the objective variable, the skin carotenoid level as the explanatory variable, and sex, academic background, current smoking and drinking status, monthly income, and age as the moderator variables.

Categorical variables were set as follows for regression analyses: sex, female = 0 and male = 1; academic background, other than university or graduate school = 0 and university or graduate school = 1; current smoking, non-smoking = 0 and smoking = 1; current drinking, non-drinking = 0 and drinking = 1; and monthly income, <10 million VND = 0 and ≥ 10 million VND = 1.

Anthropometric parameters, blood pressure, and blood parameters were converted to natural logarithms for multiple regression analysis because they did not follow a normal distribution.

BMI, body mass index; BFP, body fat percentage; SBP, systolic blood pressure; DBP, diastolic blood pressure; LDL-C, low-density lipoprotein cholesterol; HDL-C, high-density lipoprotein cholesterol; RC, regression coefficient; CI, confidence interval.

* *p* < 0.05, ** *p* < 0.01, *** *p* < 0.001.

## 4 Discussion

This cross-sectional study examined the relationship between skin carotenoid and MetS biomarker levels. The results showed that higher skin carotenoid levels were associated with lower MetS risk counts. Additionally, skin carotenoid levels were significantly negatively associated with body weight, BMI, blood glucose levels, and blood triglyceride levels. To our knowledge, this study is the first to show a relationship between carotenoid levels and the risk of MetS in Vietnam.

The mean skin carotenoid levels in the participants of this study were 5.41 ± 1.31 for all participants (*n* = 278), 4.99 ± 1.10 for males (*n* = 105), and 5.67 ± 1.37 for females (*n* = 173). The results of a cross-sectional study conducted in Japan (18) showed a mean skin carotenoid level of 5.41 ± 1.30 for all participants (*n* = 811), 4.78 ± 1.08 for males (*n* = 340), and 5.87 ± 1.25 for females (*n* = 471). The mean skin carotenoid levels in another cross-sectional study conducted in Japan (19) were 5.3 ± 1.3 for males (*n* = 604) and 6.2 ± 1.3 for females (*n* = 1,014). These results indicate that the skin carotenoid levels in Vietnamese individuals measured in this study were similar to those in Japanese individuals. Although reports on blood carotenoid concentrations in Vietnamese individuals are limited, evidence suggests that the proportion of lutein is higher in blood carotenoid concentrations of Vietnamese preschool children (27) than that in Japanese adults (18). Furthermore, a previous study on carotenoid intake in Vietnamese people (21) reported higher proportions of lutein and *β*-carotene, and a lower proportion of lycopene, compared to those observed in Japanese individuals (22, 28). The distribution of carotenoids to the skin varies depending on the type; for example, *α*-carotene and β-carotene are more readily deposited in the skin (18). Based on these reports, the skin carotenoid levels in Vietnamese and Japanese individuals may be affected by different carotenoids.

A meta-analysis of 18 studies in Vietnam showed that the prevalence of central obesity was 17.3%, that of elevated blood pressure was 24.4%, that of increased blood glucose was 16.4%, that of high triglycerides was 33.3%, and that of low high-density lipoprotein cholesterol was 34.1% (3). In this study, the prevalence of overweight/obesity was 29.5%, elevated blood pressure was 59.7%, elevated fasting blood glucose was 41.0%, elevated blood triglycerides was 44.6%, and reduced blood HDL-C was 25.5%, indicating that the prevalence of MetS components, except for reduced blood HDL-C, was higher than in a previous report. Although the criteria used to assess MetS components differed slightly between this study and the previous report, the participants in this study were likely at a higher risk of MetS than the general Vietnamese population, as they were recruited from individuals visiting a clinic.

This cross-sectional study showed that higher skin carotenoid levels are associated with lower MetS risk counts. This is consistent with the results of previous Japanese cross-sectional studies, suggesting that skin carotenoid levels are associated with a lower risk of MetS (18, 19). A higher academic background was also associated with lower MetS risk counts in this study. A previous cross-sectional study showed that the prevalence of MetS is higher in individuals with lower educational levels (29). The biological basis for the association between educational disparities and MetS remains unclear, although it has been suggested that socioeconomic status influences nutrition and sedentary habits, which are highly related to MetS components (30).

In a cross-sectional study conducted in Japan (18), higher skin carotenoid levels were significantly associated with lower BMI, SBP, DBP, blood triglyceride levels, and higher blood HDL-C levels in women, as well as significantly correlated with BMI in men. Per another Japanese cross-sectional study, higher skin carotenoid levels were associated with lower mean values of BMI, waist circumference, DBP, and serum triglycerides; serum HDL-C levels also increased with increasing skin carotenoid levels (19). Although this study did not perform a sex-specific analysis, higher skin carotenoid levels were significantly associated with lower BMI, blood glucose, and triglyceride levels, as well as marginally significantly associated with higher blood HDL-C levels. A significant negative correlation between skin carotenoid levels and blood glucose levels was noted compared to previous cross-sectional studies in Japan. A cross-sectional study in Japan reported that blood lutein concentrations showed a stronger negative correlation with blood glucose levels than with other blood carotenoid concentrations, particularly in women (8). Differences in the types of carotenoids consumed between Vietnamese and Japanese people may have influenced these results because the proportion of lutein in carotenoid intake is higher in Vietnam than in Japan, as mentioned above.

The possible mechanisms underlying the significant association between skin carotenoid levels and risk of MetS in the present study should be discussed. As skin carotenoid levels strongly correlate with blood carotenoid concentrations (18), higher skin carotenoid levels reflect higher blood carotenoid concentrations. The primary benefits of carotenoids can be explained by their antioxidant capacity (5). The risk factors associated with MetS, such as obesity, diabetes, dyslipidemia, hypertension, and impaired glucose tolerance, are characterized by the persistence of oxidative stress-mediated chronic inflammatory conditions (31). These reports suggest that carotenoids that accumulate in the body may be associated with reducing the risk of MetS by acting as antioxidants. In addition, since the proportion of lutein and *β*-carotene in carotenoid intake is higher in Vietnam, as mentioned above, the effects of these individual carotenoids should also be considered. Several human intervention studies have reported that intake of foods containing *β*-carotene reduces blood triglyceride levels (32–34), and the mechanism is thought to be related to its effects on adipocyte differentiation and lipid metabolism (35–38). Several human and animal studies have shown that lutein improves lipid metabolism (39, 40). The negative correlation between skin carotenoid levels and blood triglyceride concentrations observed in the present study may be related to the effects of β-carotene and lutein on lipid metabolism.

This study had some limitations. First, although this study included indicators such as academic background and monthly income as confounding factors, the influence of other confounding factors, such as dietary lifestyle and regular exercise, could not be ruled out. Therefore, the overall healthy lifestyle habits related to high carotenoid intake, and not only carotenoid intake itself, might have affected the results. Second, there is a possibility of selection bias as this study only included visitors to one clinic in Hanoi. A meta-analysis examining the prevalence of MetS and its related factors in Vietnam showed that living in urban areas was associated with an increased likelihood of having MetS and suggested that studies recruiting participants only in urban areas were more likely to have a higher effect size of MetS prevalence than those conducted in both urban and rural areas (3). This may be because the target population had a higher risk of MetS than the general population. Third, the association between MetS risk and individual carotenoids is unclear because this study did not measure individual blood carotenoid concentrations. It should be clarified, considering that Vietnamese and Japanese people consume different types of carotenoids. Finally, a causal relationship between carotenoid intake and MetS could not be established, as this was a cross-sectional study.

Studies considering the limitations presented above are required. First, observational studies including data on lifestyle habits closely related to MetS, such as exercise and diet, as confounding factors are important. Second, multi-center studies including institutions in rural areas outside of Hanoi are important, considering selection bias and generalizing the results of this study. Third, analyzing the association between individual blood carotenoid concentrations and the risk of MetS is important, considering that Vietnamese and Japanese individuals consume different types of carotenoids. Finally, clarifying the causal relationship between carotenoid intake and MetS through interventional studies on foods containing carotenoids is important.

## 5 Conclusion

This cross-sectional study showed that higher skin carotenoid levels are associated with lower MetS risk counts. Additionally, skin carotenoid levels were significantly negatively associated with body weight, BMI, blood glucose, and blood triglyceride levels. Although the causal relationship between carotenoid intake and MetS could not be clarified as this was a cross-sectional study, to the best of our knowledge, this study is the first to show a relationship between carotenoid levels and risk of MetS in Vietnam. Verifying the effect of carotenoid intake on MetS through intervention studies and the relationship between the risk of MetS and individual carotenoids are important future challenges.

## Data Availability

The raw data supporting the conclusions of this article will be made available by the corresponding author on reasonable request.
