# Supplementary material for: Higher skin carotenoid levels are associated with lower risks of metabolic syndrome: a cross-sectional study in Vietnamese participants
Source: Front Nutr. 2026 Jan 22;12:1715158. doi: 10.3389/fnut.2025.1715158 (PMC12872504; doi:10.3389/fnut.2025.1715158)
Supplement: Supplementary file 1 [file Data_Sheet_1.pdf]

ID：________

Questionnaire

Question 1: What is you gender? Please check the number from below.

1. ☐ Male
2. ☐ Female
3. ☐ No answer

Question 2: What is your age? Please answer your age.

Age__________

Question 3 (optional): What is your household monthly income? Please check the number from below.

1. ☐ ~10 million VND
2. ☐ 10~15 million VND
3. ☐ 15~20 million VND
4. ☐ 20~ million VND

Question 4: What is your academic background? Please check the number from below.

1. ☐ Junior high school
2. ☐ High school
3. ☐ Junior university
4. ☐ University
5. ☐ Graduate school
6. ☐ Technical school
7. ☐ Others

Question 5: Are you receiving treatment with medication under the guidance of a doctor? Please check the number from below.

1. ☐ YES
2. ☐ NO

Question 6: If you choose “1. YES” at the question 5, what kind of medicine are you taking? Please check the number from below (multiple answers allowed) and write the name of medicine.

1. ☐ For obesity
2. ☐ For diabetes
3. ☐ For hypertension
4. ☐ For hyperlipidemia
5. ☐ For hyper uricemia
6. ☐ Others

Medicine____

Question 7: Are you receiving treatment with diet therapy under the guidance of a doctor? Please check the number from below.

1. ☐ YES
2. ☐ NO

Question 8: If you choose “1. YES” at the question 7, what kind of diet therapy are you taking? Please check the number from below (multiple answers allowed).

1. ☐ Carbohydrate restriction
2. ☐ Fat restriction
3. ☐ Salt restriction
4. ☐ Increase of vegetables intake
5. ☐ Intake of supplements (vitamins, minerals)
6. ☐ Others

Question 9: Are you a smoker? Please check the number from below.

1. ☐ YES
2. ☐ NO

If yes, how many cigarettes you smoke a day?_________

Question 10: Do you drink alcohol? Please check the number from below.

1. ☐ YES
2. ☐ NO

If yes, how is the frequency of drinking (days/week).________

Question 11: How often do you eat green and yellow vegetables such as pumpkin, tomatoes, and leafy vegetables? Please check the number from below.

1. ☐ Hardly ever eat
2. ☐ Eat 1 or 2 days a week
3. ☐ Eat 3 or 4 days a week
4. ☐ Eat almost every day
